# Hydroxyurea treatment is not significantly associated with reduced spermatogonial numbers in patients with sickle cell disease

**DOI:** 10.3389/fendo.2026.1979188

**Published:** 2026-10-08

**Authors:** Ellen De Beer, Emily Delgouffe, Pauline Mazilier, Ellen Goossens

**Affiliations:** 1 Biology of the Testis Laboratory, Research Group Genetics, Reproduction and Development, Vrije Universiteit Brussel (VUB), Brussels, Belgium; 2 Hemato-oncology Department, Hôpital Universitaire des Enfants Reine Fabiola (HUDERF), Brussels, Belgium

**Keywords:** fertility preservation, hydroxyurea, pre-pubertal testicular tissue, sickle cell disease, spermatogonial stem cells

## Abstract

Hydroxyurea (HU) is considered a first-line therapy for patients with sickle cell disease (SCD). In cases of inadequate response to HU therapy, patients may receive conditioning therapy prior to haematopoietic stem cell transplantation, which can lead to depletion of the number of spermatogonia. Therefore, in pre-pubertal boys, fertility is preserved through cryopreservation of testicular tissue, although this is an experimental method. Analysis of this tissue showed a considerable loss of spermatogonia, leading to the hypothesis that HU therapy may reduce the spermatogonial number. However, evidence remains limited and conflicting. Therefore, this study aims to investigate the relationship between HU exposure and the spermatogonial number in boys with SCD. The number of spermatogonia per round seminiferous tubule (S/T) and the percentage of tubules containing spermatogonia were determined in cryopreserved testicular tissue from 27 HU-treated patients with SCD. Linear regression analyses were performed to assess their association with HU exposure. Spermatogonia were present in most boys with SCD, although in severely reduced numbers. S/T Z-scores and the percentage of tubules containing spermatogonia were not significantly associated with age at HU initiation, duration of HU exposure, or HU dose. In conclusion, in this cohort, no statistically significant association was observed between HU treatment and reduced spermatogonial numbers in patients with SCD.

## 1 Introduction

Sickle cell disease (SCD) is an autosomal recessive disorder caused by a mutation in the β-globin gene, resulting in the production of abnormal sickle haemoglobin (HbS). Following deoxygenation, HbS molecules polymerise into bundles, causing red blood cells to become sickle-shaped. The presence of these sickle-shaped red blood cells leads to chronic haemolysis and vaso-occlusion (1). Hydroxyurea (HU) is considered a first-line therapy for patients with SCD due to its ability to increase foetal haemoglobin (HbF) production. HbF inhibits the polymerisation of HbS, resulting in a reduced formation of sickle-shaped red blood cells (2).

Adults with SCD show impaired semen parameters due to vaso-occlusive episodes causing testicular hypoxia and infarctions, endocrine disruption, and oxidative stress-induced damage (3, 4). Semen parameters may further deteriorate following treatment with HU (3, 5). Sewaralthahab et al. reported that HU treatment was associated with a reduced sperm count, but semen volume, sperm motility, and sperm morphology remained unaffected (6). Notably, in adults, impairment of semen parameters appears to be related to the duration of HU exposure (7). However, in patients with a severe SCD genotype, improvements have been observed following treatment discontinuation (3, 8). In cases of inadequate response to HU therapy, patients are considered for haematopoietic stem cell transplantation (HSCT). Prior to HSCT, conditioning therapy is administered to destroy bone marrow cells and suppress the immune system, enabling donor stem cell engraftment. However, this conditioning therapy can deplete the number of spermatogonia and subsequently cause infertility (9). Because men with SCD already have reduced semen parameters, sperm banking should be considered prior to HU therapy or HSCT (4).

Both the National Heart, Lung, and Blood Institute and British Society for Haematology guidelines recommend early initiation of HU therapy from around 9 months of age in children with SCD (10, 11). Since spermatogenesis only starts at puberty, pre-pubertal boys are not eligible for sperm banking prior to gonadotoxic treatment. Given that spermatogonial stem cells are present from birth, surgical retrieval and cryopreservation of pre-pubertal testicular tissue represents a promising fertility preservation strategy for them (9). However, as this method is still experimental and HU therapy is not considered to cause a high risk for infertility, fertility preservation for pre-pubertal patients is only considered prior to HSCT.

Analysis of pre-pubertal testicular tissue has shown a reduced number of spermatogonia in patients with SCD treated with HU compared to healthy boys (12, 13). This finding is supported by Benninghoven-Frey et al., who reported that initiation of HU before the age of 2.4 years was associated with reduced spermatogonial numbers in patients with severe SCD before undergoing HSCT (14). However, Gille et al., who also analysed testicular tissue from patients with severe SCD before undergoing HSCT, compared spermatogonial numbers in HU-exposed and non-exposed patients and found no significant differences (15). Overall, current evidence regarding the effect of HU on the number of spermatogonia remains limited and conflicting. Available studies are based on a small study population of boys treated with HU, and only one study has evaluated the effect of age at HU treatment initiation and HU dose on the spermatogonial number. Given the limited literature, it remains unclear whether earlier treatment initiation, longer exposure duration, and higher doses are associated with a reduction of the spermatogonial number.

To address this gap, 27 boys diagnosed with SCD who received HU therapy were included in this study. To the best of our knowledge, this is the largest study of HU-treated patients in which cryopreserved tissue was analysed to assess the spermatogonial number. Given the potential impact of HU therapy on the number of spermatogonia, this study aims to investigate the relationship between HU exposure and the spermatogonial number in boys with SCD. Specific focus will be placed on the associations with age at treatment initiation, duration of exposure, and administered dose. These insights will enable clinicians to better counsel patients and their parents about the potential risk of reduced spermatogonial numbers associated with HU therapy.

## 2 Material and methods

### 2.1 Ethical approval

The Ethical Review Board of Universitair Ziekenhuis Brussel (B.U.N. 143201630620, B.U.N. 143201732183) and Hôpital Universitaire des Enfants Reine Fabiola (CEH n° 40/17) approved this study. This study was conducted in accordance with the principles of the Declaration of Helsinki.

### 2.2 Patient information

Testicular tissue was obtained from patients who faced gonadotoxic conditioning therapy before HSCT and wanted to preserve their fertility. At the time of biopsy, all patients were receiving HU therapy. Written informed consent was obtained from all parents and, if the patients were Gillick competent, also from the patients.

Information on HU treatment included age at treatment initiation, treatment duration, and daily dose, as documented in the patients’ medical records. The HU doses may vary depending on the progression of the disease and the patient’s growth. Older patient records included only the initial dose of HU, while more recent HU treatment records also include dose adjustments over time. When dose adjustments over time were documented, the mean dose was calculated.

### 2.3 Histological evaluation

Pre-pubertal testicular tissue was cryopreserved as part of a fertility preservation program. A small biopsy was fixed in alcohol formaldehyde acetic acid (AFA0060AF59001; VWR, Leuven, Belgium) for 1 h, followed by overnight fixation in formalin and paraffin embedding. The tissue was sectioned at a thickness of 5 µm.

Immunohistochemical staining was performed using melanoma-associated antigen A4 (MAGE-A4), which is expressed in spermatogonia and primary spermatocytes. The sections were deparaffinised in xylene (CL00.2401.5000; Analytichem, Zedelgem, Belgium), followed by a rehydration process using isopropanol-water mixtures with decreasing concentrations (100%, 100%, 90%, 70%). After washing with phosphate buffered saline (PBS; 70011051; Thermo Fisher Scientific, Breda, The Netherlands) for 5 min, endogenous peroxidases were blocked using 0.3% hydrogen peroxide in methanol for 30 min. After washing in PBS for 5 min, antigen retrieval was performed by incubating the slides in citrate buffer (home-made, pH 6) in a 95 °C water bath for 75 min. Afterwards, a blocking step was performed using 4% normal goat serum (NGS; B304, Tebu-bio, Lier, Belgium) before adding the primary antibody MAGE-A4 (Dilution 1/200; Provided by Dr Giulio Spagnoli, University of Basel, Switzerland). After an overnight incubation in a humidified chamber at 4 °C and three washing steps with PBS, the sections were incubated with a goat anti-mouse horse radish peroxidase-labelled secondary antibody (Dilution 1/100; Dako Real EnVision Detection System; K5007; Agilent Technologies, Santa Clara, CA, USA) at room temperature for 1 h. After washing three times in PBS, the staining was visualised using 3, 3′-diaminobenzidine (Dako Real Envision Detection System; K5007; Agilent Technologies, Santa Clara, CA, USA). After counterstaining with haematoxylin (made in-house), the sections underwent a dehydration process, involving isopropanol-water mixtures with increasing concentrations (70%, 90%, 100%, 100%), followed by xylene. The slides were mounted using acrytol mounting medium (3801720; Leica, Brussels, Belgium) for microscopic evaluation. The slides were analysed using an inverted microscope (CKX5381; Olympus, Aartselaar, Belgium). Images were captured using the imaging software Toupview at a magnification of 20x. The number of analysed depths per patient ranged from 3 to 18, with a minimum space of 15 µm between depths. Only one depth was available for patients 16 and 25. Spermatogonia were quantified using ImageJ software (National Institutes of Health, USA). The spermatogonial number was evaluated as the number of spermatogonia per round tubule (S/T value), which has been proposed as a standard method (13, 16). The Z-score was subsequently calculated using age-specific S/T values derived from simulated controls (17). In addition, the percentage of seminiferous tubules containing spermatogonia was assessed.

### 2.4 Statistics

Normality of the data was assessed using the Shapiro-Wilk test. Because a single outlier in the data may produce a significant test result in small sample sizes, visual inspection of histograms was also performed to assess the data distribution. Both the HU dose and duration of HU treatment showed a statistically and visually normal distribution. Statistical significance is defined as p < 0.05. However, age at HU treatment initiation indicated a statistically significant deviation from normality (P = 0.028), while visual inspection showed a normal distribution.

Linear regression analyses were performed to assess the association of S/T Z-scores or the percentage of tubules containing spermatogonia with age at initiation of HU therapy, duration of HU exposure, and daily HU dose. Combined effects of age at initiation of HU therapy, treatment duration, and/or daily dose were also assessed. The β coefficient represents the relationship between the Z-scores or tubules containing spermatogonia and HU treatment, with larger absolute values indicating stronger effects. Subsequently, scatterplots were generated to visualise these relationships. All analyses were conducted using SPSS (Version 31.0.0.0 [117]; IBM Corp., Armonk, NY, USA).

## 3 Results

The median age of the study population was 8.4 years (range 2.4 – 12.4). The median age at initiation of HU therapy was 3.0 years (range 0.7 – 9.0), with a treatment duration of 3.8 years (range 0.3 – 10.7). The median HU dose was 23.1 mg/kg/day (range 11.3 – 33.0). The study population showed a wide variation in both age and HU treatment (**Supplementary Table 1**).

The median percentage of seminiferous tubules containing spermatogonia was 41% (range 0 – 89) and the median Z-score was -1.05 (range -2.27 – 1.31). Among the 27 pre-pubertal samples, 18 patients had S/T values below previously published reference ranges. Two patients did not have any spermatogonia (**Supplementary Table 1**). These low S/T values were also visualised by the scatter plot, with 25 negative Z-scores indicating S/T values below the reference mean and 2 positive Z-scores indicating S/T values above the reference mean (**Figures 1A-C**).

**Figure 1:**
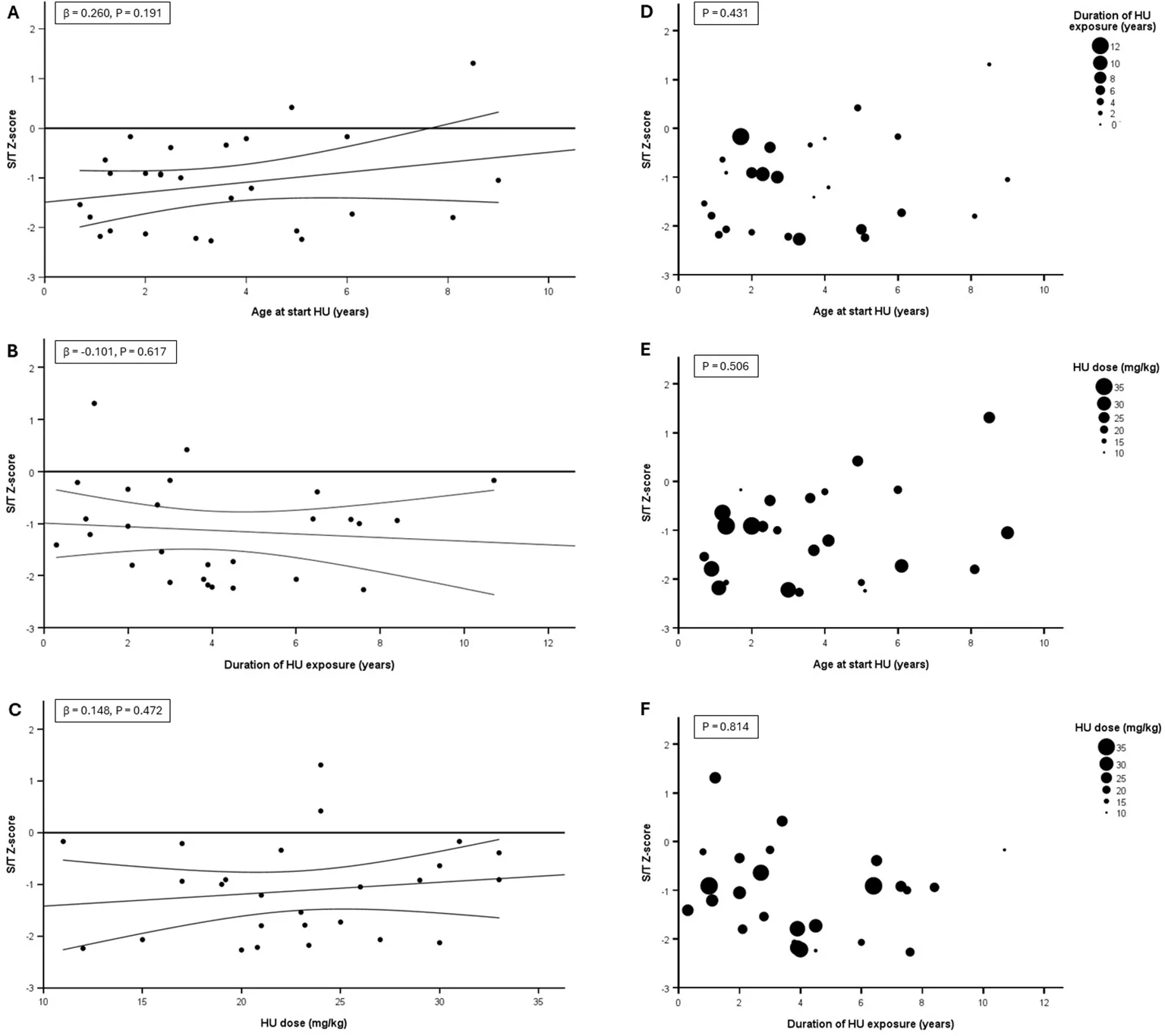
Impact of hydroxyurea (HU) exposure on the number of spermatogonia per round tubule (S/T) in patients with sickle cell disease. Graphical display of S/T Z-scores in relation to age at initiation of HU therapy **(A)**, duration of HU exposure **(B)**, HU dose **(C)**, age at initiation of HU therapy with duration of HU exposure **(D)**, age at initiation of HU therapy with HU dose **(E)**, and duration of HU exposure with HU dose **(F)**. Regression lines with 95% confidence intervals are shown in panels **(A–C)**.

In this study population, no significant associations were observed between Z-scores and age at initiation of HU therapy (β = 0.260, 95% CI [-0.054, 0.255], P = 0.191, R^2^ = 0.067), duration of HU exposure (β = -0.101, 95% CI [-0.176, 0.107], P = 0.617, R^2^ = 0.010), or HU dose (β = 0.148, 95% CI [-0.042, 0.088], P = 0.472, R^2^ = 0.022) (**Figures 1A-C**). Furthermore, no significant associations were found between Z-scores and the following combinations: age at initiation of HU therapy with duration of HU exposure (P = 0.431), age at initiation of HU therapy with HU dose (P = 0.506), duration of HU exposure with HU dose (P = 0.814) (**Figures 1D-F**), and age at initiation of HU therapy with duration of HU exposure with HU dose (P = 0.714). Similarly, no significant associations were found between the percentage of seminiferous tubules containing spermatogonia and age at HU initiation (β = 0.215, 95% CI [-2.3, 7.6], P = 0.281, R^2^ = 0.046), duration of HU exposure (β = 0.004, 95% CI [-4.465, 4.546], P = 0.985, R^2^ = 0.000), or HU dose (β = 0.059, 95% CI [-1.797, 2.385], P = 0.774, R^2^ = 0.003) (**Figures 2A-C**). Moreover, no significant associations were found between the percentage of seminiferous tubules containing spermatogonia and any of the tested combinations: age at initiation of HU therapy with duration of HU exposure (P = 0.525), age at initiation of HU therapy with HU dose (P = 0.603), duration of HU exposure with HU dose (P = 0.838) (**Figures 2D-F**), and age at initiation of HU therapy with duration of HU exposure with HU dose (P = 0.802).

**Figure 2:**
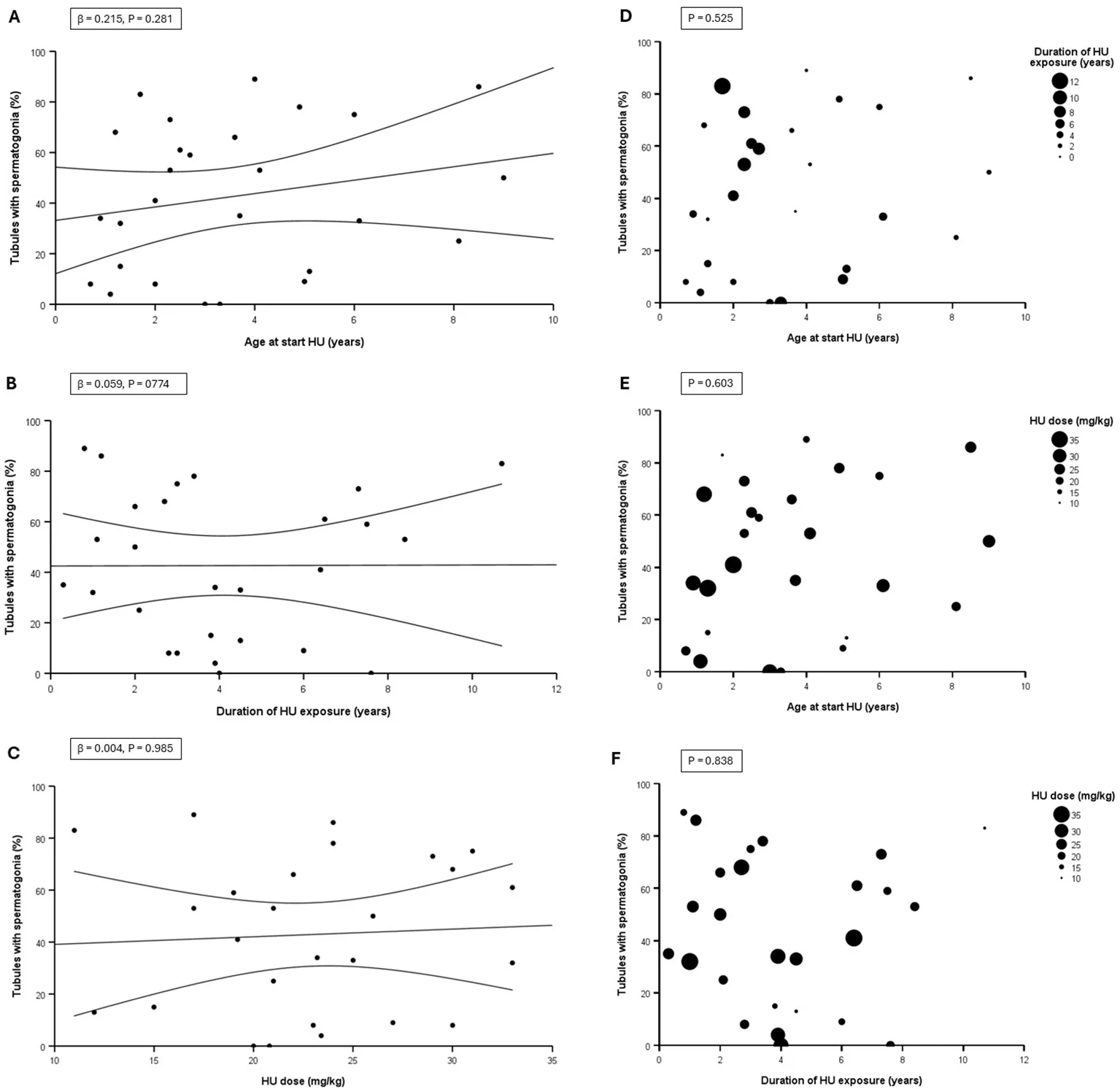
Impact of hydroxyurea (HU) exposure on the percentage of seminiferous tubules with spermatogonia in patients with sickle cell disease. Graphical display of tubules with spermatogonia in relation to age at initiation of HU therapy **(A)**, duration of HU exposure **(B)**, HU dose **(C)**, age at initiation of HU therapy with duration of HU exposure **(D)**, age at initiation of HU therapy with HU dose **(E)**, and duration of HU exposure with HU dose **(F)**. Regression lines with 95% confidence intervals are shown in panels **(A–C)**.

## 4 Discussion

Analysis of cryopreserved pre-pubertal testicular tissue has shown reduced spermatogonial numbers in patients with SCD treated with HU compared to healthy boys, with HU initiation before the age of 2.4 years being associated with lower spermatogonial numbers (12–14). However, Gille et al. found no significant differences in spermatogonial numbers between HU-exposed and non-exposed patients (15). Current evidence regarding the effect of HU on the number of spermatogonia remains limited and conflicting, raising the question whether earlier treatment initiation, longer exposure duration, and higher doses are associated with a reduction in the spermatogonial number. Therefore, this study aimed to investigate the relationship between HU exposure and the spermatogonial number in boys with SCD, with specific focus on age at treatment initiation, duration of exposure, and administered dose.

In this study, spermatogonia were present in the testicular tissue of most boys with SCD, although in severely reduced numbers. However, no statistically significant associations were found between the spermatogonial number and age at initiation of HU therapy, duration of HU exposure, or HU dose. Therefore, these findings may suggest that the observed reduction in the spermatogonial number is mainly caused by SCD itself. This may be explained by the fact that patients with SCD experience recurrent vaso-occlusive episodes, which can lead to testicular chronic hypoxia and infarctions, inflammation, and oxidative stress-induced damage (4, 18) These injuries may induce germ cell apoptosis and ultimately impair spermatogenesis (4). In addition, altered spermatogonial DNA methylation in pre-pubertal patients with SCD has also been suggested as a potential contributing mechanism, as correct DNA methylation is essential for spermatogonial maintenance (19). These findings do not support routine fertility preservation before HU treatment in pre-pubertal boys. However, fertility preservation may be considered as early as possible after SCD diagnosis. Patients with SCD may experience repeated testicular infarctions (18), which could potentially lead to a progressive reduction in the number of spermatogonia with each infarction.

To the best of our knowledge, the present study includes the largest cohort of pre-pubertal boys with SCD treated with HU to date, exceeding the cohorts described by Benninghoven-Frey et al. and Gille et al., which included 21 and 17 HU-treated patients, respectively. Compared with our study population, Benninghoven-Frey et al. included older patients, patients who received higher HU doses, and patients who had a shorter duration of HU exposure. The age at HU initiation was comparable between both populations. In both studies, patients were receiving HU at the time of biopsy. Benninghoven-Frey et al. counted spermatogonia in round seminiferous tubules following immunohistochemical staining for MAGE-A4. Nevertheless, their study evaluated more tubules than the present study for the assessment of the S/T Z-score. Benninghoven-Frey et al. reported significant differences in spermatogonial numbers following HU initiation before the age of 2.4 years. In the present study, 11 patients started HU treatment before the age of 2.4 years. The median age of these patients at initiation of HU therapy was 1.3 years (range 0.7 – 2.3) with a treatment duration of 3.9 years (range 1 – 10.7), the median HU dose was 23.2 mg/kg/day (range 11.3 – 33), and the median age at the time of biopsy was 5 years (range 2.4 – 12.4). In contrast to the findings reported by Benninghoven-Frey et al., no significant differences in spermatogonial numbers were observed following earlier initiation of HU therapy. Consistent with these, no association was found between HU dose and a reduced spermatogonial number (14). Compared with the study population reported by Gille et al., the present study included younger patients, patients who initiated HU treatment at a younger age, a wider range of HU doses, and a substantially longer duration of HU exposure. Gille et al. performed immunohistochemical staining for MAGE-A4 and evaluated more seminiferous tubule cross-sections than in the present study. Gille et al. compared spermatogonial numbers in HU-exposed and non-exposed patients and found no significant differences. However, it should be noted that 11/17 patients underwent testicular biopsy after a median HU washout period of 5.2 months, which may have limited their ability to detect statistically significant differences between HU-exposed and non-exposed patients (15).

A major limitation of this study is the absence of HU non-exposed patients with SCD. Therefore, it is not possible to distinguish the effects of HU therapy from the underlying disease process. As HU is the first-line therapy for SCD, most patients are already exposed before undergoing HSCT, making it difficult to include a HU non-exposed control group. Therefore, it was not possible to assess the role of SCD in the reduction of the spermatogonial number. Nevertheless, significantly reduced semen parameters in patients with SCD not exposed to HU suggest that SCD causes impaired spermatogenesis (20, 21). However, HU treatment in adulthood further impairs semen parameters by inducing S-phase arrest in proliferating germ cells (22). Another limitation of this study is that only patients with severe SCD were included, as these patients required HSCT for which they banked testicular tissue. The increased incidence of testicular infarctions in patients with severe SCD may contribute to the observed depletion of spermatogonial numbers. Therefore, these results cannot be extrapolated to patients with mild SCD. Moreover, the greater testicular damage observed in patients with severe SCD represents a potential confounding factor when assessing the effect of HU exposure. In addition, the relatively small sample size (n = 27) may limit the strength of the conclusions, as the G*Power analysis indicated that a sample size of 55 patients would be required. Consequently, the absence of statistically significant associations between HU exposure and spermatogonial number does not exclude HU-related toxicity or establish SCD itself as the principal cause of spermatogonial depletion. Furthermore, spermatogonial numbers do not directly establish future reproductive capacity. For three patients, HU dose adjustments over time were documented and the average dose was calculated. Cumulative HU exposure, which takes both dose and treatment duration into account, may be a more relevant measure than average dose. However, as cumulative HU exposure was only available for three patients, it was not possible to assess their association with spermatogonial numbers.

Future studies should include a larger study population, as well as a wider range of age at HU treatment initiation, duration of exposure, and administered dose to better assess the impact of HU exposure on the spermatogonial number. In addition, the inclusion of HU non-exposed patients would be useful to distinguish the effects of SCD and HU therapy on the spermatogonial number. However, cryopreserved testicular tissue from HU non-exposed patients is currently not available, as HU represents the standard first-line treatment for SCD and is therefore routinely initiated before cryopreservation.

In this cohort, no statistically significant association was observed between HU treatment and reduced spermatogonial numbers in patients with SCD.

## Data Availability

The dataset supporting the clinical outcomes of this study is securely stored in the Vrije Universiteit Brussel (VUB) Institutional Data Repository under restricted access, with the accession number VUB/GRAD/1/000022, to protect participant privacy. Access requests will be reviewed on an individual basis and must be directed to Prof. Ellen Goossens (ellen.goossens@vub.be). She will assess the request, considering the research purpose and potential commercial applications. Prior to any data sharing or release, a data use agreement, in compliance with the VUB legal department's guidelines, must be completed and signed. Metadata for the datasets is available through the VUB Research Portal at: https://researchportal.vub.be/en/datasets/spermatogonial-stem-cell-pool-in-boys-with sickle-cell-disease-tr/.
