# Supplementary material for: Hydroxyurea treatment is not significantly associated with reduced spermatogonial numbers in patients with sickle cell disease
Source: Front Endocrinol (Lausanne). 2026 Oct 8;17:1979188. doi: 10.3389/fendo.2026.1979188 (PMC13686923; doi:10.3389/fendo.2026.1979188)
Supplement: Supplementary file 1 [file Table1.docx]

**Supplementary Table 1. Spermatogonial stem cell pool in boys with sickle cell disease treated with hydroxyurea.**

| **Patient** | **Age at biopsy (years)** | **Age at start HU (years)** | **Duration of HU exposure (years)** | **HU dose (mg/kg/d)** | **Tubules with spermatogonia (%)** | **S/T value** | **Reference S/T value ^a^** | **Z-score** |
| --- | --- | --- | --- | --- | --- | --- | --- | --- |
| 1 | 3.5 | 0.7 | 2.8 | 22.9 | 8 | **0.11** | 2.0 (1.7 – 2.3) | -1.54 |
| 2 | 4.8 | 0.9 | 3.9 | 23.2 | 34 | **0.47** | 2.0 (1.7 – 2.3) | -1.79 |
| 3 | 5.0 | 1.1 | 4.0 | 23.4 | 4 | **0.05** | 2.5 (2.1 – 2.9) | -2.18 |
| 4 | 3.9 | 1.2 | 2.7 | 30.4 | 68 | **1.32** | 2.0 (1.7 – 2.3) | -0.64 |
| 5 | 5.1 | 1.3 | 3.8 | 15.3 | 15 | **0.16** | 2.5 (2.1 – 2.9) | -2.07 |
| 6 | 2.4 | 1.3 | 1.0 | 33.0 | 32 | **0.95** | 1.2 (1.0 – 1.4) | -0.91 |
| 7 | 12.4 | 1.8 | 10.7 | 11.3 | 83 | 6.00 | 5.0 (2.0 – 8.0) | -0.17 |
| 8 | 8.4 | 2.0 | 6.4 | 19.2 | 41 | **1.45** | 2.5 (1.9 – 3.1) | -0.91 |
| 9 | 5.0 | 2.0 | 3.0 | 30.0 | 8 | **0.10** | 2.5 (2.1 – 2.9) | -2.13 |
| 10 | 11.7 | 2.2 | 8.4 | 17.3 | 53 | 2.57 | 5.0 (2.0 – 8.0) | -0.94 |
| 11 | 9.6 | 2.3 | 7.3 | 28.5 | 73 | **1.43** | 2.4 (1.8 – 3.1) | -0.92 |
| 12 | 9.0 | 2.6 | 6.5 | 33.0 | 61 | 2.00 | 2.4 (1.8 – 3.1) | -0.39 |
| 13 | 10.1 | 2.7 | 7.5 | 19.0 | 59 | **1.35** | 2.5 (1.9 – 3.1) | -1.00 |
| 14 | 7.0 | 3.0 | 4.0 | 20.8 | 0 | **0** | 2.6 (2.2 – 3.1) | -2.22 |
| 15 | 10.9 | 3.3 | 7.6 | 20.0 | 0 | **0** | 2.5 (1.9 – 3.1) | -2.27 |
| 16 | 5.5 | 3.6 | 2.0 | 22.0 | 66 | **2.03** | 2.5 (2.1 – 2.9) | -0.34 |
| 17 | 4.0 | 3.7 | 0.3 | NK | 35 | **0.88** | 2.0 (1.7 – 2.3) | -1.41 |
| 18 | 4.7 | 4.0 | 0.8 | 16.8 | 89 | 2.17 | 2.5 (2.1 – ­2.9) | -0.21 |
| 19 | 5.2 | 4.1 | 1.1 | 21.2 | 53 | **1.09** | 2.5 (2.1 – 2.9) | -1.21 |
| 20 | 8.1 | 4.9 | 3.4 | 24.0 | 78 | 2.86 | 2.5 (1.9 – 3.1) | 0.42 |
| 21 | 10.1 | 5.0 | 6.0 | 27.0 | 9 | **0.22** | 2.4 (1.8 – 3.1) | -2.07 |
| 22 | 9.6 | 5.1 | 4.5 | 12.0 | 13 | **0.03** | 2.4 (1.8 – 3.1) | -2.24 |
| 23 | 8.9 | 6.0 | 3.0 | 31.0 | 75 | 2.23 | 2.4 (1.8 – 3.1) | -0.17 |
| 24 | 10.6 | 6.1 | 4.5 | 25.0 | 33 | **0.58** | 3.3 (1.0 – 5.6) | -1.73 |
| 25 | 10.2 | 8.1 | 2.1 | 20.9 | 25 | **0.50** | 2.5 (1.9 – 3.1) | -1.80 |
| 26 | 9.6 | 8.5 | 1.2 | 24.0 | 86 | 3.80 | 2.4 (1.8 – 3.1) | 1.31 |
| 27 | 10.3 | 9.0 | 2.0 | 25.9 | 50 | **1.30** | 2.4 (1.8 – 3.1) | -1.05 |

HU = hydroxyurea, NK = not known

^a^ Reference values for spermatogonial numbers are reported by Masliukaite *et al.*(16)

Spermatogonial numbers in bold indicate values below the reference
